# Compulsive sexual behavior disorder: rates and clinical correlates in a community sample

**DOI:** 10.3389/fpsyt.2025.1561885

**Published:** 2025-05-22

**Authors:** Jon E. Grant, Sophie Boutouis, Madison Collins, Samuel R. Chamberlain

**Affiliations:** ^1^ Department of Psychiatry & Behavioral Neuroscience, University of Chicago, Chicago, IL, United States; ^2^ Department of Psychiatry, Faculty of Medicine, University of Southampton, Southampton, United Kingdom; ^3^ NHS Southern Gambling Service/Southern Health NHS Foundation Trust, Southampton, United Kingdom

**Keywords:** compulsive sexual behavior, compulsivity, hypersexual disorder, impulsivity, sex, sexual addiction

## Abstract

**Background:**

This study sought to examine the rate of compulsive sexual behavior disorder (CSBD) in a sample of adults drawn from the community and its associated mental health correlates.

**Methods:**

An online survey of “Personality, Mental Health, and Well-Being” was distributed via Prolific to 300 adults aged 18 to 75 years. The survey measured a range of behaviors, such as sexual behavior, alcohol and drug use, and dimensional constructs of impulsivity and compulsivity using validated self-report instruments.

**Results:**

A total of 296 participants (54.7% female) completed the survey and were included in the analysis. The overall prevalence of probable compulsive sexual behavior disorder (CSBD) was 10.8% (*n*=32). Compared to adults without CSBD, those with CSBD were more likely to be younger and identify as bisexual. In addition, they were more likely to have attention-deficit hyperactivity disorder (ADHD) and borderline personality disorder (BPD) symptoms, social media and drug use problems, and higher levels of obsessionality, trans-diagnostic compulsivity, and trans-diagnostic impulsivity. Gender, race, and alcohol use did not significantly differ between groups.

**Conclusion:**

CSBD appears to be common in adults and is equally represented in males and females. CSBD appears to have obsessive, compulsive, and impulsive traits and this may have nosological importance.

## Introduction

Although mentioned in the medical literature for well over a hundred years under various names, and recently included in the International Classification of Diseases Version 11 (ICD-11), Compulsive Sexual Behavior Disorder (CSBD) continues to be a contested diagnostic entity (1, 2). The generally agreed upon understanding of CSBD is that it reflects some level of difficulty controlling sexual urges and behaviors and that this lack of control results in clinically significant distress and/or psychosocial impairment (3). There is ongoing debate, however, as to whether the urges and behaviors associated with CSBD are best understood as an addiction (such as gambling), an impulse control disorder (such as kleptomania), or as lying somewhere on the obsessive-compulsive spectrum (e.g., 4–6).

One way to understand behaviors that are poorly controlled, and to determine the most appropriate classification for them, is to examine the potential underlying trans-diagnostic constructs of impulsivity and compulsivity. Impulsivity refers to the tendency to choose the immediacy of reward despite possible negative consequences (i.e. that there is something rewarding/pleasurable driving the behavior and that this urge for reward outweighs the consequences); whereas compulsivity is the tendency to perform repetitive habitual actions largely to reduce an unwanted anxious feeling, despite the resultant psychosocial impairment (7). These concepts of impulsivity and compulsivity, however, may not be diametrically opposed driving forces behind human behavior (8). In the case of CSBD, some research has begun addressing these constructs (9–13) as well as understanding CSBD by examining comorbidity and its longitudinal course (14, 15).

In a small sample of people with CSBD (*n*=20), researchers found elevated rates of impulse control disorders (ADHD) as well as elevated rates of impulsivity using the Barratt Impulsiveness Scale (16). Impulsivity in at least two areas was one of the most observed BPD symptoms in a sample of 85 patients with CSBD, though only one patient met criteria for BPD (17). Müller and Antons (18) examined 102 adult males and found heightened impulsivity (defined as urgency and deficits in perseverance) in the subset of adults with high levels of problematic pornography use (problematic pornography use has been argued to be a major subtype of CSBD; 19). Similarly, Mestre-Bach et al. (20) examined 44 adults with CSBD who were seeking treatment and found that they scored higher on the obsessive-compulsive subscale of the Symptom Checklist-Revised compared to adults with gambling disorder or those with internet gaming disorder. Although these studies found both impulsive (i.e. reward-driven) and compulsive (i.e. habitual and ritualistic) traits in adults with CSBD, other research has found that compulsivity and impulsivity may only play modest roles in problematic sexual behavior (21).

Because the data regarding impulsive and compulsive traits in CSBD are sparse, we conducted a small exploratory study of individuals in the community who met proposed criteria for CSBD compared with healthy controls. The study hypothesized that those with probable CSBD would endorse greater levels of both trans-diagnostic impulsivity and trans-diagnostic compulsivity.

## Methods

The Department of Psychiatry and Behavioral Neuroscience at the University of Chicago developed the Personality, Mental Health, and Well-Being Survey to assess mental health and well-being in a large sample of adults online. The survey included demographic information and used questions from validated screening tools examining mental health issues. All study procedures, including the consent procedure, were carried out in accordance with the Declaration of Helsinki and were approved by the Institutional Review Board of the University of Chicago.

Participants completed the online survey via REDCap (REDCap is a secure web platform managed by the University of Chicago) as part of this study. Participants were first required to view the Institutional Review Board (IRB) – approved consent page, at which point they could choose to participate in the survey or opt out. A refusal to respond was taken as a denial of consent and participants were not allowed to continue with the study. The survey asserted that all responses would be kept confidential and that no personally identifying information would be collected. Subjects were compensated $12 for their participation. Data was collected on 1/9/24.

The self-report survey took approximately 30 minutes to complete. Survey questions assessed demographic information (including self-identified gender, race, educational attainment, and sexual orientation, in which respondents could choose from various categorical options), sexual behavior, and mental health and substance use issues (especially traits and symptoms relevant to impulsivity and compulsivity).

The following reliable and valid measures were embedded (without titles) in the survey: the Minnesota Impulsive Disorders Interview (MIDI) (22, 23) (the Compulsive Sexual Behavior Disorder [CSBD] module reflects urges, fantasies and behaviors as well as impairment and distress and was based on phenomenological research regarding CSBD) (24, 25); the Alcohol Use Disorders Identification Test (AUDIT) (26) (to examine the oftentimes impulsive behavior of alcohol use; the AUDIT demonstrated excellent reliability in the present study [α = .846]); the Drug Abuse Screening Test (DAST-10) (27, 28) (to examine rates of drug use which is often seen as impulsive; the DAST-10 demonstrated good reliability in the present study [α = .633]); the Adult ADHD Self-Report Scale Part A (ASRS-v1.1) (29) (a disorder with prominent impulsive features and large prevalence rates; the ASRS demonstrated good reliability in the present study [α = .761]); the McLean Screening Instrument for Borderline Personality Disorder (MSI-BPD) (30) (another disorder with pronounced impulsive features and large prevalence rates; the MSI-BPD showed excellent reliability in the present study (α = .943); the Barratt Impulsiveness Scale, Short Form (BIS-15) (31) (the BIS-15 demonstrated excellent reliability in the present study [α = .869]); the Cambridge-Chicago Compulsivity Trait Scale (CHI-T) (32, 33) (a scale examining compulsivity/rigidity of behaviors; the CHI-T demonstrated excellent reliability in the present study [α = .811]); the Bergen Social Media Addiction Scale (BSMAS) (34) (the BSMAS demonstrated excellent reliability in the present study [α = .867]); the Problematic Tinder Use Scale (PTUS) (35) (the PTUS demonstrated good reliability in the present study [α = .782]); and the Obsessive-Compulsive Inventory Revised (OCI-R) (36) (a scale of a more prototypical compulsive behavior; the OCI-R demonstrated excellent reliability in the present study [α = .896]).

Subjects screened positive for CSBD on the MIDI if they reported any of the following: 1) an intense preoccupation with some aspect of sex or excessive sexual activity; 2) repetitive sexual fantasies or urges that are out of control or cause significant distress; or 3) engagement in repetitive sexual behavior that is out of control or causes significant distress.

Four participants did not complete the CSBD module of the MIDI, bringing the final sample size to 296. Some participants did not complete every clinical measure in the survey (resulting in different *N*s for each outcome variable displayed in **Table 1**). The PTUS could only be completed by participants who reported current dating app use, which is why the *N* for the PTUS is much smaller than the other measures.

### Data analysis

A sample of 300 adults aged 18 to 75 years were sent the online survey via Prolific. Assuming an expected prevalence of CSBD of around 10% (e.g. 37), this would yield a CSBD group of ~30 individuals and a reference group of ~270. This would yield 95% power to detect a significant group difference of large effect size (d=0.7), at alpha 0.05, two-tailed (G*power software).

Participants were grouped into one of two categories based on their responses to the CSBD module of the MIDI (see above for diagnostic criteria) with respect to the last 12 months: probable CSBD and No CSBD. Significant main effects of group were identified for demographic measures using independent sample *t* tests for continuous variables and chi-square tests or fisher’s exact tests for categorical variables. Since age was significantly correlated with the total scores of the clinical measures, analyses of covariance (ANCOVAs) were used to control for age while detecting significant between-group differences. Effect sizes were calculated in the forms of Cohen’s d for *t* tests (0.2 = small, 0.5 = medium, 0.8 = large), Phi for chi-square tests (0.1 = weak, 0.3 = moderate, 0.5 = strong), and partial eta squared for ANCOVAs (0.01 = small, 0.06 = medium, 0.14 = large). Finally, we created a binary logistic regression model in which various dimensions of impulsivity and compulsivity (i.e., ASRS, BIS-15, CHI-T, MSI-BPD, OCI-R total scores) were examined as possible predictors of probable CSBD (0 = no, 1 = yes) while adjusting for age and sexual orientation (0 = not bisexual, 1 = bisexual). Variance Inflation Factor (VIF) values were calculated to evaluate the predictor variables for multicollinearity. The VIF values in our study ranged from 1-3, suggesting moderate correlations between the predictors but no multicollinearity. The fit of the model was verified with the Hosmer-Lemeshow test. Our sample SPSS was used for all statistical analyses (version 24; IBM Corp). Statistical significance was defined as *p* < 0.05.

## Results

The demographics for the sample who completed the CSBD module of the MIDI (*n*=296) are presented in **Table 1**. The overall prevalence of probable compulsive sexual behavior disorder (CSBD) was 10.8% (*n*=32) based on a screening questionnaire (not on a diagnostic assessment). Among females, the rate of probable CSBD was 10.1%, and the rate was 12.3% among males.

**Table 1:** Descriptive statistics for a sample of 296 adults.

Demographics		*M (SD)* or %
Age in years Gender	Female	37.47 (12.62) 54.7
	Male Other	42.2 3.1
Race	White or Caucasian	69.4
	Black or African American	14.1
	Hispanic or Latino	5.2
	Asian or Pacific Islander Other	3.4 7.9
Educational attainment	Some high school	1.4
	High school Some college College Master’s degree Professional degree	12.3 26.0 47.3 11.6 1.4
Sexual orientation	Heterosexual	78.2
	Gay	1.7
	Lesbian	1.7
	Bisexual Other	12.6 5.8
Current nicotine use	Yes No	17.7 82.3

**Table 2** shows the demographics of the adults with probable CSBD compared to the No CSBD participants. Those with probable CSBD were significantly younger (*p*=0.004) and more likely to identify as bisexual (*p*=0.014).

**Table 2:** Descriptive statistics for a sample of 296 adults, stratified by a positive versus negative CSBD screen.

Demogreaphics		Group		*t* or Pearson Chi-Square	*p*	Cohen’s d or Phi
		Negative CSBD screen (*n* = 264) *M (SD)* or %	Positive CSBD screen (*n* = 32) *M (SD)* or %			
Age in years Gender	Female	38.21 (12.69) 55.3	31.44 (10.44) 50.0	2.899 0.329	0.004 0.848	0.543 0.034
	Male Other	41.6 3.1	46.9 3.1			
Race	White or Caucasian	70.7	59.4	3.105	0.540	0.103
	Black or African American	13.1	21.9			
	Hispanic or Latino	5.0	6.3			
	Asian or Pacific Islander	3.1	6.3			
	Other	8.1	6.3			
Educational attainment	Some high school	1.5	0	9.869	0.079	0.184
	High school Some college College Master’s degree Professional degree	10.4 27.3 47.7 11.5 1.5	28.1 15.6 43.8 12.5 0			
Sexual orientation	Heterosexual	80.1	62.5	12.472	0.014	0.206
	Gay	1.9	0			
	Lesbian	1.5	3.1			
	Bisexual	10.3	31.3			
	Other	6.1	3.1			
Current nicotine use	Yes	16.8	25.0	1.319	0.251	0.067
	No	83.2	75.0			

Those with probable CSBD scored significantly higher on multiple mental health measures (**Table 3**). They were more likely to have problematic social media use and drug use problems (*p*<.001). They reported higher scores on a measure of obsessive-compulsive symptoms (OCI-R) (*p*<.001) and compulsivity (CHI-T) (*p*=0.018). Finally, they scored higher on the BIS-15 (*p*=0.005), a measure of impulsivity, and had more severe symptoms of ADHD (p<.001) and BPD (p<.001), disorders with prominent impulsive features.

**Table 3:** Total scores on a variety of clinical measures for a sample of adults, stratified by a positive versus negative CSBD screen.

Assessments	Group		*F*	*p*	
	Negative CSBD screen N=264 *M (SD)* or %	Positive CSBD screen N=32 *M (SD)* or %			Partial eta squared
BSMAS PTUS	246 11.88 (4.69) 21 11.52 (2.50)	32 15.97 (5.08) 9 13.78 (4.21)	16.264 3.387	<.001 0.077	0.056 0.111
OCI-R	246 13.45 (10.09)	32 24.03 (12.25)	25.822	<.001	0.086
MSI-BPD	254 2.40 (2.52)	32 5.81 (3.02)	41.896	<.001	0.129
ASRS-5	253 7.30 (4.12)	32 11.00 (3.87)	18.269	<.001	0.061
AUDIT	252 3.54 (4.40)	32 4.59 (4.85)	1.767	0.185	0.006
DAST-10	258 0.50 (1.14)	32 1.56 (2.29)	15.948	<.001	0.053
CHI-T	245 23.11 (6.24)	31 26.26 (5.63)	5.652	0.018	0.020
BIS-15	249 29.54 (7.29)	30 34.07 (7.38)	7.979	0.005	0.028

Age was controlled for while assessing differences in all total scores between groups.

BSMAS, Bergen Social Media Addiction Scale.

PTUS, Problematic Tinder Use Scale.

OCI-R, Obsessive Compulsive Inventory – Revised.

MSI-BPD, McLean Screening Instrument for BPD.

ASRS-5, Adult ADHD Self-Report Scale for DSM-5.

AUDIT, Alcohol Use Disorders Identification Test.

DAST-10, Drug Abuse Screening Test.

CHI-T, Cambridge-Chicago Compulsivity Trait Scale.

BIS, Barratt Impulsiveness Scale.

The results of the logistic regression revealed that greater ADHD (OR = 1.20, *p* = .028) and BPD (OR = 1.25, *p* = .041) symptomology increased the odds of having probable CSBD, even after controlling for age, sexual orientation, and other measures of impulsivity and compulsivity. See **Table 4**.

**Table 4:** Logistic regression predicting probable CSBD (yes versus no).

Predictor	B	SE (B)	Odds ratio	95% CI	*t*	*p*
Age	-.018	.023	.982	.939, 1.026	.664	.415
Bisexual	.738	.540	2.092	.726, 6.026	1.869	.172
OCI-R	.023	.026	1.023	.972, 1.077	.765	.382
MSI-BPD	.224	.110	1.251	1.009, 1.551	4.186	.041
ASRS-5	.179	.082	1.196	1.019, 1.403	4.798	.028
DAST-10	.219	.127	1.245	.970, 1.597	2.969	.085
CHI-T	-.003	.044	.997	.914, 1.088	.003	.953
BIS-15	-.062	.044	.940	.863, 1.024	1.992	.158
Constant	-2.605	1.634	0.074		2.542	.074

The results of the logistic regression revealed that greater ADHD and BPD symptomology increased the odds of meeting criteria for CSBD, even after controlling for age, sexual orientation, and other measures of impulsivity and compulsivity.

OCI-R, Obsessive Compulsive Inventory – Revised.

MSI-BPD, McLean Screening Instrument for BPD.

ASRS-5, Adult ADHD Self-Report Scale for DSM-5.

AUDIT, Alcohol Use Disorders Identification Test.

DAST-10, Drug Abuse Screening Test.

CHI-T, Cambridge-Chicago Compulsivity Trait Scale.

BIS-15, Barratt Impulsiveness Scale.

## Discussion

We examined prevalence of probable CSBD in a sample of adults in the community and found a prevalence rate of 10.8%, with females equally represented among those with probable CSBD. Interestingly, this rate of 10.8% is akin to the percentage of people reported to have distress linked to difficulty controlling sexual thoughts/behaviors in a large US sample (*n*=2,325) (37), but is somewhat higher than the typical rates reported in previous CSBD work (often 3-6%) (38) or in a large international study (4.8%) (39). Because probable CSBD was linked to younger age in our study, one possible explanation for the variation in the literature could be the age of a particular sample examined or to the measure used to assess for the disorder.

In terms of the mental health issues among those with probable CSBD, these adults reported addictive problems, issues with trans-diagnostic impulsivity (BIS-15 scores) and more trans-diagnostic compulsivity (CHI-T scores) than those without CSBD. Thus, these transdiagnostic measures suggest that probable CSBD may not be simply categorized as an addictive, impulsive, or a compulsive issue as these adults with probable CSBD reported elevated rates of problems across these contextual domains. This may highlight a wider issue with current classification systems – namely that disorders are by necessity put into a particular category, but a potential disadvantage of this approach is that other features inherent in the given condition may then tend to be overlooked clinically. One useful approach to complement this categorical approach may be to additionally consider trans-diagnostic measures such as trait impulsivity and compulsivity.

There are several implications of these findings. Probable CSBD appears to be fairly common, yet in many parts of the world no treatment services exist, which should perhaps be rectified (40). Probable CSBD was linked to elevated rates of problematic usage of social media. The link here may be the wider umbrella construct of Problematic Usage of the Internet (PUI) (for discussion see: 41, 42). Perhaps PUI constitutes the link underlying the relationship between probable CSBD (which can manifest through compulsive online activities), and other forms of PUI. This could be addressed in future work by measuring the extent to which probable CSBD manifests online versus off-line. Clinically, it may be useful to incorporate measures of PUI into practice as part of evaluations. The elevated levels of impulsive and compulsive problems in people with probable CSBD highlights the need for careful clinical assessment for other related impulsive-compulsive disorders, such as (but not limited to) ADHD, BPD, and obsessive-compulsive related disorders.

While this study measured trans-diagnostic impulsivity and compulsivity in probable CSBD in one study setting, several limitations should be noted. This study was not designed to be epidemiologically representative and so findings may not generalize to other settings. The gold standard for diagnosis is of course structured clinical interviews – whereas this study used previously validated self-report instruments. The MIDI CSBD module used herein to identify probable CSBD is not identical to the ICD-11 criteria for CSBD, since the former was developed at an earlier time point. As such, any diagnoses should be considered as provisional/likely but not certain in relation to ICD-11. Additionally, some concepts examined are not formal disorders but rather concepts such as problematic use of Tinder or social media. This being an exploratory study we did not correct for multiple comparisons; this had the benefit of reducing the risk of false negatives due to the power arising from the given sample size of the probable CSBD group. Relatedly, the sample size was relatively small, and while power was ample to detect large effect size group differences, ability to detect more subtle group differences (i.e. those of small or medium effect size) would have been limited. We did not examine all possible measures that may reflect impulsivity and compulsivity. We relied only on self-report instruments. Future work might wish to expand on the measures used – for example to include cognitive measures. Another important limitation is that impulsivity and compulsivity can be considered in the broad sense (i.e. trans-diagnostically, such as using BIS-15 and CHI-T) as well as at the level of how a particular behavior manifests phenomenologically. For example, one individual may undertake compulsive sexual behavior in the spur of the moment in order to obtain a reward; and thus, the behavior may be described as being impulsive; while another person may undertake such behavior in a repetitive fashion to alleviate anxiety or according to rigid rules, thus being compulsive. Or both may apply, to differing degrees, depending on the person and the point in time. In the current study we focused on trans-diagnostic impulsivity and compulsivity measured cross-sectionally, and did not collect behavior-specific measures. Behavior-specific impulsivity and compulsivity could potentially be measured in a variety of ways in future work – such as asking a given person about subjective drivers underlying specific behaviors (e.g. via a questionnaire); and/or using cognitive tests that attempt to model behavioral responses to stimuli under laboratory conditions. This type of approach may also be useful to better understand how CSBD should optimally be classified.

In conclusion, this study identified relatively high rates of probable CSBD in an online sample and that it was linked to elevated rates of impulsive and compulsive problems and traits. This has implications for clinical practice but also highlights the need for further research into CSBD including how its presentation may change longitudinally and interact with PUI and other clinical features.

## Data Availability

The raw data supporting the conclusions of this article will be made available by the authors, without undue reservation.
